# Splicing factor SRSF2-centric gene regulation

**DOI:** 10.7150/ijbs.58888

**Published:** 2021-04-16

**Authors:** Kun Li, Ziqiang Wang

**Affiliations:** 1 Department of Nuclear Medicine, The First Affiliated Hospital of Shandong First Medical University & Shandong Provincial Qianfoshan Hospital, Jinan 250014, China.; 2 Biomedical Sciences College & Shandong Medicinal Biotechnology Centre, Shandong First Medical University & Shandong Academy of Medical Sciences, Jinan 250062, China.

**Keywords:** SRSF2, SC35, gene regulation, transcription, splicing, mRNA stability.

## Abstract

Serine/arginine-rich splicing factor 2 (SRSF2) is a splicing factor that is widely expressed in a variety of mammalian cell types. Increasing evidence has confirmed that SRSF2 plays vital roles in a number of biological and pathological processes. Therefore, it is important to understand how its expression is regulated, and how it regulates the expression of its target genes. Recently, we found that SRSF2 expression could be upregulated by herpes simplex virus-1 (HSV-1) infection*,* and that altered SRSF2 expression, in turn, epigenetically regulates the transcription of HSV-1 genes. Further studies on T cell exhaustion demonstrated that upregulated SRSF2 in exhausted T cells elevated the levels of multiple immune checkpoint molecules by associating with the acyl-transferases, P300 and CBP, and by altering histone modification near the transcription start sites of these genes, thereby influencing signal transducer and activator of transcription 3 binding to these gene promoters. These findings suggest that SRSF2 acts as an important sensor and effector during disease progression. Here, we discuss the molecules that regulate *SRSF2* gene expression and their associated mechanisms, and the mechanisms via which SRSF2 regulates the expression of target genes, thus providing novel insights into the central role of SRSF2 in gene regulation.

## Introduction

Serine/arginine-rich splicing factor 2 (SRSF2), previously known as SC35, is a member of a well-known serine/arginine-rich (SR) protein family. SRSF2 is an important component of the nuclear structure, speckle. It consists of an RNA recognition motif (RRM) and a domain rich in arginine and serine residues (RS domain). SRSF2 has been shown to control the splicing of pre-mRNAs by recognizing and binding to SRSF2-binding sites on pre-mRNA via the RRM and by interacting with other SR splicing factors via the RS domain 1,2. Unlike other SR splicing factors, SRSF2 has a longer L3 loop region and prefers to recognize highly degenerated RNA sequences of exonic splicing enhancers (ESEs) 3,4. However, recent studies have found that SRSF2 is also involved in regulating genomic stability, gene transcription, mRNA stability, and translation 5-8.

Increasing evidence has demonstrated that SRSF2 and other SR proteins are associated with the progression of a variety of diseases, including viral infection and tumorigenesis. Majerciak et al. reported that serine/arginine-rich splicing factor 3 (SRSF3) interacted with open reading frame 57 (ORF57) to mediate splicing of Kaposi sarcoma-associated herpesvirus (KSHV) K8β RNA via RNA binding motif protein 15 (RBM15) and OTT3 (RBM15B), a close member to RBM15 9. Our previous study of the interaction between host cellular factors and herpes simplex virus-1 (HSV-1) found that SRSF2 enhances HSV-1 replication and viral gene expression by epigenetically regulating the transcription of viral genes 5. Jacquenet et al. reported that SRSF2 modulates human immunodeficiency virus type 1 (HIV-1) replication by downregulating the levels of HIV-1 structural proteins and genomic RNA 10, and by mediating the splicing of *Tat* 11-14 and *Rev* 15, which are required for HIV-1 replication. In another study, we investigated the role of SRSF2 in T cell exhaustion during tumorigenesis and demonstrated that SRSF2 expression is significantly elevated in exhausted T cells, and that SRSF2 knockdown reverses the exhaustion of T cells by epigenetically regulating the expression of multiple immune checkpoint molecules 6. In addition, an increasing number of studies have recently shown that *SRSF2* gene mutation and dysregulated SRSF2 expression are significantly associated with acute myeloid leukemia (AML) 16, 17, myelodysplastic syndromes 18-20, systemic mastocytosis 21, 22, chronic myelomonocytic leukemia 23-25, lung carcinoma 26, 27, hepatocellular carcinoma 28, and sinonasal squamous cell carcinoma 29. These findings indicate that SRSF2 functions as a promising therapeutic target for various diseases.

In this review, we discuss the molecules that regulate SRSF2 expression, and their associated regulatory mechanisms. These molecules are mainly transcription factors, miRNAs, protein kinases, and acetyltransferases that can influence SRSF2-mediated gene regulation by altering the transcriptional and post-transcriptional levels and the modification of SRSF2 protein. We also discuss the molecular mechanisms via which SRSF2 regulates the expression of its target genes.

## Factors and regulatory patterns of SRSF2 expression and protein modification

To date, many factors have been found to regulate *SRSF2* gene expression and modification of SRSF2 protein. Some factors influence *SRSF2* gene transcription, pre-mRNA splicing, and mRNA translation. Others have been shown to phosphorylate, acetylate, or deacetylate SRSF2 protein. These factors, which are summarized in Table 1, are generally transcriptional factors, miRNAs, protein kinases, and acetyltransferases.

### Transcription

E2F transcription factor 1 (E2F1) is a transcriptional regulator of *SRSF2*. As an important trans-activator of pro-apoptotic target genes, E2F1 has been shown to regulate the alternative splicing of various apoptotic genes, including FADD-like apoptosis regulator (*c-Flip*), caspases-8 (*Casp8*) and -9 (*Casp9*), and *Bcl-x*, by increasing *SRSF2* gene transcription. Molecular mechanism investigation showed E2F1 directly targeted the promoter region 296-79 bp upstream of *SRSF2* to initiate its transcription, which is required for apoptosis in response to drugs that induce DNA damage 30.

### Alternative splicing

*SRSF2* mRNAs consist of different 3' untranslated regions (UTRs) and exhibit significantly different stabilities 31 and the splicing of *SRSF2* pre-mRNA has been shown to be autoregulated by SRSF2. SRSF2 enhances both exon inclusion and intron excision in the 3' UTR of the *SRSF2* pre-mRNA, resulting in a significant decrease in endogenous *SRSF2* mRNA levels and an increase in the levels of alternatively spliced SRSF2 mRNAs, both of which have a short half-life 32. Further investigation showed that there are multiple low-affinity SRSF2-binding sites located within a highly conserved stem-loop region of the terminal exon 33.

### mRNA stability and translation

Several miRNAs have been shown to target *SRSF2* to regulate *SRSF2* mRNA stability and translation. In a study aimed at understanding the resistance of hepatocellular carcinoma to 5-fluorouracil (5-FU), the stability of *SRSF2* mRNA, the splicing regulator of caspase 2, which contributes to tumor apoptosis in response to 5-FU, was found to be decreased by DNA methylation-regulated *miR-193a-3p* 34*.* Another investigation of the resistance of bladder cancer to pirarubicin, paclitaxel, adriamycin, and epirubicin hydrochloride found that *miR-193a-3p* targeted *SRSF2* mRNA to repress its expression levels, which then activated Notch and oxidative stress, two chemoresistance-associated signaling pathways 35. In addition to chemoresistance, *miR-193a-3p*-regulated *SRSF2* has been shown to be involved in radio-resistance. *miR-193a-3p* was found to increase the resistance of nasopharyngeal cancer cells to radiation by targeting *SRSF2* and thus, activating the hypoxia signaling pathway 36. Moreover, two miRNAs, *miR-183-5p* and *miR-33a-5p*, were found to contribute to the upregulation of SRSF2 expression by methylxanthine caffeine by targeting *SRSF2* and repressing the translation of SRSF2 37.

### Protein modification

Growing evidence has shown that the splicing activity of SRSF2 and other SR proteins are affected by its phosphorylation. SRSF protein kinase 1 (SRPK1) 38, phosphatidylinositol 3-kinase (PI3K)/Akt pathway 39, 40, and PH domain and leucine rich repeat protein phosphatase 1/2 (PHLPP1/2) 41 were found to regulate splicing by mediating the phosphorylation and dephosphorylation of SR proteins, respectively. SRSF2 is an important splicing regulator of *tau* pre-mRNA 8, and the dysregulation of its splicing often results in neurodegenerative disorders 42, 43. To date, many factors have been reported to regulate *tau* pre-mRNA splicing by mediating the phosphorylation and acetylation of SRSF2. Glycogen synthase kinase-3β, a serine-threonine kinase that functions as a regulator of pre-mRNA processing through the phosphorylation of splicing factors 44,45, has been shown to interact with and phosphorylate SRSF2, resulting in the enrichment of SRSF2 in nuclear speckles and the loss of its ability to mediate splicing events 46. This contributes to aberrant tau splicing 47. Moreover, another protein kinase, dual-specificity tyrosine-phosphorylated and regulated kinase 1A (Dyrk1A), also regulates SRSF2-mediated splicing of *tau* pre-mRNA. Dyrk1A suppresses the ability of SRSF2 to promote *tau* exon 10 inclusion, by interacting with the N-terminus of SRSF2 and phosphorylating SRSF2 48. However, the phosphorylation of SRSF2, regulated by protein kinase A (PKA) 49 and HIV-1 Tat 50, enhances SRSF2-mediated *tau* exon 10 inclusion. In addition, the deacetylase, sirtuin-1, inhibits the SRSF2-promoted *tau* exon 10 inclusion by interacting with and deacetylating SRSF2 51.

In addition to the splicing ability of SRSF2, modifications of SRSF2 protein influence its turnover. The acetyltransferase, Tip60, has been found to acetylate SRSF2 on lysine 52 in the RRM, resulting in the proteasomal degradation of SRSF2, whereas histone deacetylase 6 counters this acetylation to function as a positive regulator of SRSF2 protein levels. Moreover, Tip60 inhibits SRSF2 phosphorylation by downregulating the nuclear levels of SRPK1 and SRPK2 52, two serine/arginine protein kinases specific for the SR domain family 38, 53.

## Regulation of gene expression by SRSF2

SRSF2 has been reported to be involved in the regulation of gene transcription, pre-mRNA splicing, mRNA transport, and mRNA stability. In this section, we will discuss the mechanisms by which SRSF2 regulates its target genes (Table 2).

### Transcription

Our previous study investigating the role of SRSF2 in HSV-1 infection found that SRSF2 facilitated HSV-1 viral replication and gene expression by binding to viral gene promoters and associating with RNA polymerase II, infected cell protein 27 (ICP27), and infected cell protein 8 (ICP8), to promote gene transcription 5. In another study investigating the role of SRSF2 in T cell exhaustion, we found that SRSF2 expression is upregulated in exhausted T cells and the inhibition of SRSF2 downregulates the transcription of many immune checkpoint genes, which contributes to dysfunctional cytokine secretion by T cells, cells proliferation, tumor cell cytotoxicity, and effective memory cell generation 54, through associating with the acetyltransferase CBP/P300 complex, altering histone modifications, and thereby recruiting the transcriptional factor signal transducer and activator of transcription 3 to the promoter of immune checkpoint genes to initial gene transcription 6.

In addition, SRSF2 acts as a transcriptional regulator during cell cycle progression. SRSF2 has been shown to be a cell cycle-related protein that is involved in regulating entry and progression into S phase, by associating with the transcription factor, E2F1, and recruiting it to the promoters of *Skp2* or cyclin E, to upregulate the transcription of these cell cycle-related genes 55.

### Alternative splicing

Alternative splicing of pre-mRNAs contributes significantly to human proteomic complexity, and aberrant alternative splicing plays a key role in the progression of various diseases. SRSF2 has been shown to be involved in the alternative splicing of many target pre-mRNAs that are associated with human diseases, including viral infection, tumors, and neurodegenerative diseases.

In our previous study, we found that SRSF2 mediated the alternative splicing of HSV-1 infected cell protein (*ICP0*) pre-mRNA by associating with *ICP0* exon 3, which contains the SRSF2-binding motif 5. In HIV-infected cells, SRSF2 profoundly changes the HIV-1 splicing pattern, in which SRSF2 mainly promotes Tat1 production by enhancing splicing at site A3, which is necessary for *Tat* mRNA synthesis 10,13. Further investigation identified a complex cis-acting element in *tat* exon 2 that is required for splicing regulation. An ESE was found to be located within the regulatory element and SRSF2 was reported to activate *tat* exon 2 splicing by binding to the ESE 14. For adenovirus E1a pre-mRNAs, SRSF2 promotes 13S production via an increase in the utilization of most downstream 5′ splicing sites 56. However, for simian vacuolating virus 40 (SV40) early pre-mRNA, SRSF2 overexpression resulted in a significant inhibition of splicing 56.

Many tumor-associated genes are also regulated by SRSF2. MDM2 is a nuclear-localized E3 ubiquitin ligase that accelerates tumor formation by targeting p53, a tumor suppressor, and mediating its proteasomal degradation 57-59. A recent study investigating genotoxic stress-induced MDM2 splicing found that SRSF2 promotes the inclusion of MDM2 exon 11 by binding to two conserved ESEs located at exon 11 60.

Colony-stimulating factor 3 receptor (CSF3R), a member of the family of cytokine receptors that function to regulate the production, differentiation, and function of granulocytes 61, contains three *CSF3R* mRNA splice variants, namely V1, V3, and V4, with different expression levels. A study to examine the expression levels of these *CSF3R* mRNA variants in patients with AML found that those harboring SRSF2 mutations and SRSF2 knock-out cells both exhibit a significant alteration in the V3/V1 ratio, suggesting SRSF2-mediated *CSF3R* splicing 16.

Soluble isoforms of vascular endothelial growth factor receptors 1 (sVEGFR1) have been implicated in several physical and pathological processes, including tumorigenesis 62, 63. In lung cancer cells, SRSF2 has been found to positively regulate the expression level of sVEGFR1-i13, one of the s*VEGFR1* mRNA splice variants, with no correlation between *SRSF2* and *VEGFR1* mRNA levels, indicating that SRSF2 controls the splicing of sVEGFR1-i13 64.

Vascular endothelial growth factor A (VEGF-A), a growth factor active in angiogenesis and endothelial cell growth, is correlated with tumor progression via the formation of blood vessels 65. *VEGF-A* mRNAs mainly consists of three isoforms, namely *VEGF121*, *VEGF165*, and *VEGF189* 66*.* A study aimed at determining the roles of E2F1 and SRSF2 in VEGF-A expression and pre-mRNA splicing found that SRSF2 overexpression increased the production of the *VEGF165b* splice variant, an inhibitor of the growth of several types of tumors, by inhibiting the migration and proliferation of endothelial cells 67, resulting in a decrease in tumor neovascularization and tumor formation 68.

Ron, also known as MST1R, PTK8, or SEA, is a member of the MET proto-oncogene family 69, whose members function as inducers of tumor progression by binding to macrophage-stimulating protein, and thereby stimulating the phosphorylation on C-terminal docking sites of multiple transducer and adaptor proteins 70. By physically interacting with the CGAG sequence in exon 11, SRSF2 enhances the inclusion of exon 11 in *Ron* pre-mRNA, resulting in an increase in the production of Ron△165 71, which promotes invasive growth and metastasis 72.

CD44 is a cell membrane glycoprotein that mediates cell growth, differentiation, and motility 73. The V6 exon-containing isoforms of *CD44* mRNA (*CD44* V6) have been implicated in tumorigenesis by promoting tumor cell invasion and metastasis 74-76. SRSF2 has been shown to positively regulate the expression level of *CD44* V6 and the V6 exon and its flanking introns contain SRSF2 response elements, indicating that SRSF2 is involved in the inclusion or exclusion of the V6 exon 77.

Protein kinase C delta (PKCδ), a serine/threonine kinase, plays a key role in cell proliferation, differentiation, and apoptosis 78. A study investigating the role of retinoic acid in the splicing of *PKCδ* pre-mRNA identified that SRSF2 upregulates the expression level of PKCδVIII, a pro-survival splice variant of PKCδ, by promoting the selection of 5' splice site II and binding to an ESE in *PKCδ* exon 10 79.

Kruppel-like factor 6 (KLF6), a member of the Kruppel-like family of transcription factors, functions as a tumor suppressor and is implicated in tumorigenesis 80, 81. A study of the regulation of the alternative splicing of *KLF6* pre-mRNA by caffeine found that caffeine induced the expression of SRSF2, and upregulated SRSF2 levels promoted exon 1a inclusion in *KLF6* pre-mRNA 82.

Recent studies have also shown that SRSF2 promotes the pre-mRNA splicing of several apoptosis-related genes, including *Casp8*, *Casp9*, *c-Flip*, and *Bcl-x*, in response to DNA-damaging agents, resulting in an increase in the expression levels of proapoptotic splice forms of these apoptotic genes, and subsequently, inducing apoptosis 30,34.

In addition to genes related to virus infection and tumor progression, SRSF2 has been shown to mediate the pre-mRNA splicing of multiple neurodegenerative disease-related genes. During the pathogenesis of Alzheimer's disease (AD), disrupted alternative splicing of β-amyloid precursor protein (*APP*) and *tau* pre-mRNA has been found to contribute to the accumulation of β-amyloid peptide (Aβ) and tau, the characteristics of AD 83-85. A study of the regulation of *APP* splicing showed that SRSF2 is involved in the alternative splicing of *APP* exons 7 and 8 by binding to Alu elements on either side of exon 86. Another study of the role of SRSF2 in regulating tau expression found that SRSF2 positively regulated the expression of tau isoforms containing exon 10 by binding to this exon, suggesting that the regulation of *tau* pre-mRNA splicing by SRSF2 is dependent on exon 10 8.

In addition to the promotion of pre-mRNA splicing, SRSF2 has also been reported to directly repress exon inclusion. Studies of the splicing regulator of survival of motor neuron 2 (*SMN2*) gene, whose aberrant splicing contributes to disease severity in spinal muscular atrophy patients, by the deletion or mutation of the *SMN1* gene 87, 88, found that overexpression of SRSF2 reduces *SMN2* exon 7 inclusion by binding to a 10 nt RNA sequence surrounding the branch-point, located upstream of the 3′ splice site (3′ SS), thus promoting the selection of the 3′ SS 89-91.

### RNA stability and transport

In addition to the regulation of gene transcription and alternative splicing, SRSF2 has been reported to be involved in regulating RNA stability and transport. A study investigating the molecular mechanism by which SRSF2 promotes tau40 expression found that SRSF2 overexpression leads to the accumulation of *tau40* mRNA and prevents its degradation by actinomycin D, an inhibitor of DNA transcription and replication 92, via association with the RRM domain of *tau* exon 10 8. This indicates that SRSF2 stabilizes *tau40* mRNA.

Overexpression of SRSF2 also results in an accumulation of the unspliced *SV40* pre-mRNA, and the exporting of most of the unspliced *SV40* pre-mRNA to the cytoplasm, suggesting a role of SRSF2 in regulating RNA stability and transport 56. In addition, a study investigating the role of SRSF2 in human papillomavirus 16 gene expression found that SRSF2 knock-down decreases the expression levels of *E6E7* RNAs. A mechanistic study showed that SRSF2 maintains the stability of *E6E7* mRNAs 93.

## Conclusion

**Figure 1:**
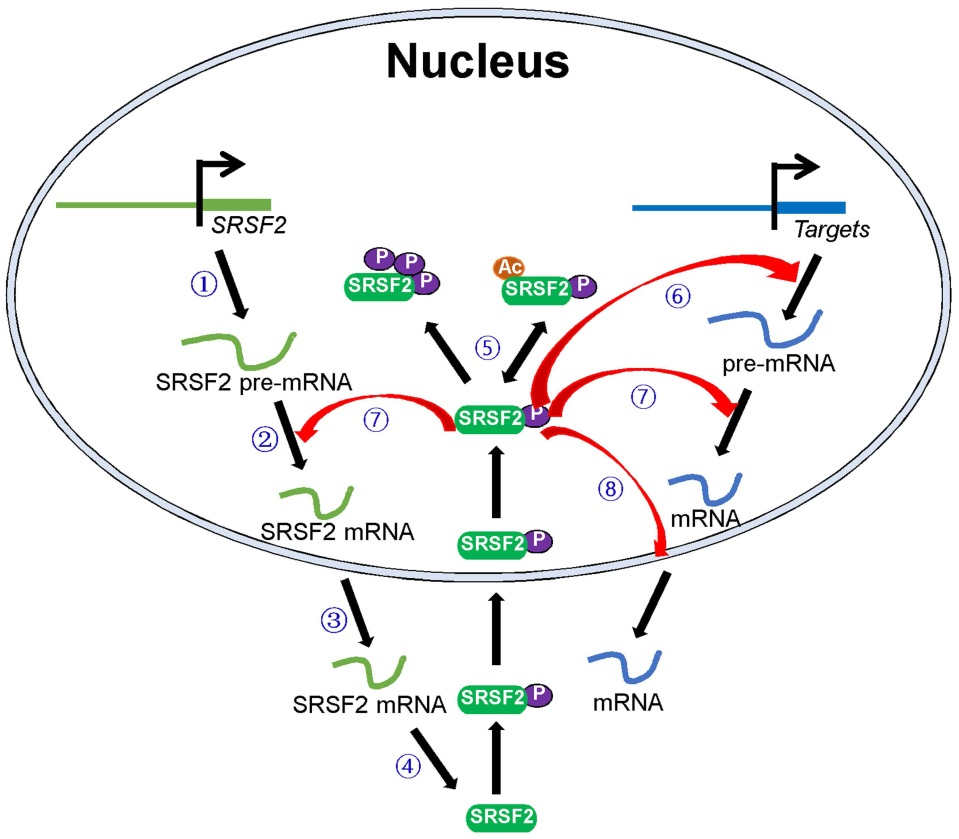
** Schematic model of the central roles of SRSF2 in genes expression.** Numbers of regulators are found to modulate SRSF2 gene transcription ①, pre-mRNA splicing ②, mRNA stability ③, mRNA translation ④, and SRSF2 modification (phosphorylation and acetylation) ⑤. In turn, SRSF2 regulates target gene transcription ⑥, pre-mRNA splicing ⑦, and mRNA transport and stability ⑧.

SRSF2 mutations and dysregulated SRSF2 expression have been shown to be associated with various diseases. In this review, we focused on how these pathological processes regulate SRSF2 expression and how SRSF2 regulates these processes. In particular, we discussed the molecules that regulate SRSF2 expression and their associated mechanisms. Most of these regulatory molecules are transcription factors, miRNAs, protein kinases, and acetyltransferases. They play important roles in *SRSF2* gene transcription, pre-mRNA splicing, mRNA stability, and translation and SRSF2 protein modification. In addition, SRSF2, in turn, was found to mediate the processes via which it regulates the expression of target genes (Figure 1). Overall, this review discusses SRSF2-centric gene regulation and provides insights into its potential clinical utilities.

## Figures and Tables

**Table 1:** Factors regulating SRSF2 expression and protein modification.

Cell Type	Factor	Regulatory Pattern	Function	Reference
H358 and H1299 cells	E2F1	Transcription	Up (alternative splicing)	30
HeLa Tet-On cells	SRSF2	Alternative splicing	/	32
HeLa cells	SRSF2		/	33
SMMC-7721 and QGY-7703 cells	miR-193a-3p	mRNA stability and translation	Down (alternative splicing)	34
5637 cells and H-bc cells	miR-193a-3p		Down (alternative splicing)	35
CNE-1 and CNE-2 cells	miR-193a-3p		Down (alternative splicing)	36
HeLa cells	miR-183-5p and miR-33a-5p		/	37
HeLa and MG63 cells	SRPK1	Phosphorylation and acetylation	Down (alternative splicing)	38
DT40 cells	PI3K/Akt		Up (alternative splicing)	39
HEK293T cells	PI3K/Akt		Up (alternative splicing)	40
MEFs	PHLPP1 and 2		/	41
HeLa cells	GSK-3β		Down (alternative splicing)	46
Cortical neurons	GSK-3β		Down (alternative splicing)	47
HEK-293T cells	Dyrk1A		Down (alternative splicing)	48
HEK-293FT and HeLa cells	PKA		Up (alternative splicing)	49
Embryonic neurons	HIV-1 Tat		Down (alternative splicing)	50
HeLa and HEK-293FT cells	SIRT1		Down (alternative splicing)	51
H1299 and H358 cells	TIP60		Up (alternative splicing)	52

**Table 2:** SRSF2 regulate genes expression.

Cell type	Target	Regulatory pattern	References
HeLa cells	HSV-1 ICP0, ICP27, and TK	Transcription	5
JurkateE6.1 cells	PD-L1, BTLA, CTLA4, LAG3, and CD160		6
H1299 and HeLa cells	Skp2 and Cyclin E		55
HeLa cells	HSV-1 ICP0	Alternative splicing	5
HEK-293T and HeLa cells	Adenovirus E1a		56
HEK-293T and HeLa cells	SV40 early pre-mRNA		56
HEK-293T and HeLa cells	HIV-1 Tat		10,12,14
MCF-7 cells	MDM2		60
KG-1, HL-60 and promyelocytic cells	CSF3R		16
MGH7 cells	VEGFR1		64
H358 cells	VEGF-A		68
MDA MB 231 and HeLa cells	Ron		71
MCF-7 cells	CD44		77
H358 cells	c-Flip, Caspase -8, -9, and Bcl-x		30
SMMC-7721 and QGY-7703 cells	Caspase 2		34
NT2/D1 cells	PKCδ		79
HeLa cells	KLF6		82
HEK293 cells	APP		86
HEK-293T cells	TAU		8
HEK-293T and HeLa cells	SMN2		89-91
HEK-293T cells	TAU	RNA stability and transport	8
W12ti tumor cells	HPV16 E6E7		93
HEK-293T and HeLa cells	SV40 early pre-mRNA		56

## Abbreviations

SRSF2: Serine/arginine-rich splicing factor 2
SR: Serine/arginine-rich
RRM: RNA recognition motif
RS domain: Arginine and serine residues
ESE: Exonic splicing enhancer
ORF57: Open reading frame 57
KSHV: Kaposi sarcoma-associated herpesvirus
RBM15: RNA binding motif protein 15
HSV-1: Herpes simplex virus-1
HIV-1: Human immunodeficiency virus type 1
AML: Acute myeloid leukemia
E2F1: E2F transcription factor 1
c-FLIP: Cellular FADD-like IL-1β-converting enzyme-inhibitory protein
Casp8: Caspases-8
Casp9: Caspases-9
UTRs: Untranslated regions
5-FU: 5-Fluorouracil
SRPK1: SRSF protein kinase 1
PI3K: Phosphatidylinositol 3-kinase
Dyrk1A: Dual-specificity tyrosine-phosphorylated and regulated kinase 1A
PKA: Protein kinase A
ICP27: Infected cell protein 27
ICP8: Infected cell protein 8
ICP0: Infected cell protein 0
SV40: Imian vacuolating virus 40
CSF3R: Colony-stimulating factor 3 receptor
sVEGFR1: Soluble isoforms of vascular endothelial growth factor receptors 1
VEGF-A: Vascular endothelial growth factor A
CD44 V6: V6 exon-containing isoforms of CD44 mRNA
PKCδ: Protein kinase C delta
KLF6: Kruppel-like factor 6
AD: Alzheimer's disease
APP: Amyloid precursor protein
Aβ: β-Amyloid peptide
SMN2: Survival of motor neuron 2
3′ SS: 3′ splice site
